# Acceptance of Digital Technology Among Nursing Staff in Geriatric Long-Term Care: Systematic Review

**DOI:** 10.2196/82223

**Published:** 2026-01-15

**Authors:** Jeton Iseni, Walter Swoboda, Daniel Houben, Roman Hilla

**Affiliations:** 1 DIWAG, Health Management, University of Applied Sciences Neu-Ulm, Wileystr. 1, Neu-Ulm, 89231, Germany, +49 730780880, +49 730780860; 2 DigiHealth, Health Management, University of Applied Sciences Neu-Ulm, Neu-Ulm, Germany; 3 Faculty of Social Work, University of Applied Sciences Landshut, Landshut, Germany; 4 Business Intelligence, Administration, St. Elisabeth Pflegezentrum Senden, Senden, Germany

**Keywords:** digitalization, elderly care, health information technology, geriatric nurse, long-term care, LTC, nursing, organizational innovation, systematic review, technology acceptance

## Abstract

**Background:**

Digital technologies are increasingly being introduced into the health care system and in settings such as hospitals and geriatric long-term care (LTC) facilities, offering potential benefits such as improved care quality, reduced workload, or enhanced documentation processes. However, the success of these technologies also depends on the acceptance by the primary users, that is, the nursing staff.

**Objective:**

This review synthesizes empirical studies that have explored the acceptance of digital technologies by nursing staff in geriatric LTC settings, building upon the foundational work by Yu et al (2009). The goal is to identify influencing factors, assess the extent of existing evidence, and highlight research gaps in this care setting.

**Methods:**

A systematic literature review was conducted following PRISMA (Preferred Reporting Items for Systematic Reviews and Meta-Analyses) 2020 guidelines. The SPIDER (sample, phenomenon of interest, design, evaluation, research type) framework was used for eligibility criteria. Databases searched included PubMed, ACM Digital Library, Web of Science, and the Health Administration Database ProQuest. Studies were included if they empirically examined the acceptance of digital technologies by nursing staff in geriatric LTC settings. Two reviewers independently screened the studies, extracted data, and assessed methodological quality using the CASP (Critical Appraisal Skills Programme) checklist.

**Results:**

A total of 3 studies met the criteria, highlighting a gap in research on this topic. The studies applied cross-sectional quantitative designs and highlighted critical determinants of technology acceptance, including perceived usefulness, ease of use, digital competence, and organizational support. The studies involved a total of 1019 participants from Germany, Australia, and the Netherlands. Barriers included lack of user involvement, lack of training, poor system design, and demographic differences in digital affinity.

**Conclusions:**

This review shows that the acceptance of digital technologies by nursing staff in geriatric LTC settings is shaped by a constellation of individual factors, such as digital competence and perceived relevance of technology, as well as organizational factors such as access to training and involvement of staff in the implementation process. Despite these insights, the limited number of empirical studies highlights a research gap in this care setting. To ensure sustainable digital transformation in geriatric LTC, future research should prioritize rigorous and participatory approaches, using longitudinal, intervention-based, or multilevel study designs.

## Introduction

### Overview

“A promising approach to understanding social dynamics lies in conceiving our society as a globalized knowledge society undergoing a comprehensive and multifaceted digital transformation” [1]. The adoption of digital technologies in health care and nursing care reflects the complex digital transformation taking place across society [2]. Digital technologies are already having an immense impact on how nursing care is delivered [3-10]. In elderly care settings, particularly in geriatric long-term care (LTC) facilities, digital technologies such as electronic health records, assistive robotic systems, telehealth apps, assistive sensory systems, information and communication technologies, or artificial intelligence monitoring platforms [4,9,11] offer important opportunities to address current and future challenges [12-15]. These include workforce shortages, improving working conditions, or increasing the attractiveness of the nursing profession. The demographic shift associated with an aging population [16] is also one of the major challenges in this context. In Germany, the number of individuals in need of LTC rose to over 5.7 million people by December 2023 [17], with projections indicating a further increase in this number. In Germany, several programs were initiated for supporting the digital pathway [18,19]. The Bavarian State Chancellery decided in a cabinet meeting on March 19, 2024, to promote digitalization in health care and nursing. The goal is to further improve medical and nursing care for the population [20]. On the other hand, not only is the demand for LTC places increasing, but also the need for nursing staff in general is growing [21].

The real-world implementation of digital innovations in the health care system, especially elderly care, remains inconsistent and is frequently challenging [7,22-25]. One of the most significant challenges is the level of acceptance among nursing staff [26-29]. As the primary users of these technologies and new systems, nursing staff play a crucial role in determining whether such tools will be adopted and integrated into everyday work [15-19]. While research in acute and primary care has increasingly examined digital transformation through staff training, workflow redesign, and implementation frameworks, geriatric LTC remains comparatively underexplored. In acute care settings, digital competence programs and structured IT implementation strategies are often supported by institutional infrastructure [30,31]. Theoretical models such as the technology acceptance model (TAM) [32] or TAM2 [33] highlight that perceived usefulness and perceived ease of use are key predictors of user acceptance [34]. However, practical experience shows that digital transformation, especially in the field of care, often falters at the stage of user engagement, particularly when it fails to consider organizational, cultural, ethical, and educational conditions [2,5,10,35-38]. In geriatric LTC, where staff is more involved in basic care of older adults, these challenges become even more important [14,39-41]. A simple example of how digital technology in geriatric LTC could avoid high risks and time waste of the nursing staff is the occurrence of discrepancies between medication plans sent via fax by general practitioners and the actual administration records in nursing homes. Paper-based updates made during medical visits are sometimes not transferred into the official documentation, creating dangerous information gaps and avoidable risks for residents. This example illustrates how outdated communication practices and the lack of integrated digital infrastructures can compromise care quality and safety. It further highlights the importance of user-accepted digital solutions in daily nursing work and a scientifically grounded framework for implementation in LTC. Geriatric LTC facilities often face limited access to training resources and less technical and managerial support for digital adoption. Consequently, empirical evidence on how nursing staff in LTC acquire digital skills, engage in technology implementation, and perceive organizational support remains scarce. This gap underscores the need for research specifically focusing on acceptance factors, training needs, and contextual barriers unique to geriatric LTC, rather than extrapolating findings from hospital-based studies. Despite the critical role of nursing staff in implementing digital innovations, scientific evidence addressing their perspectives, needs, and acceptance in LTC contexts remains very low [25,42].

### Objective

Despite considerable political interest and investments in digital transformation, the success of such efforts in the care setting hinges on a crucial factor that remains underexplored, at least in the geriatric LTC, which is the acceptance of digital technologies by nursing staff. Their perspective is not only relevant but essential to the sustainable implementation of digital solutions in care. The primary objective of this systematic review is to synthesize existing empirical research that investigates the acceptance of digital technologies among nursing staff in geriatric LTC settings, building upon the work of Yu et al [39], which was one of the first studies with focus on acceptance factors among nursing staff in LTC, published in 2009. By identifying the most relevant influencing factors, the review contributes to a better understanding of the conditions under which circumstances digital innovations can be effectively and successfully implemented in geriatric LTC environments, with particular attention to the acceptance factors of the nursing staff in this setting.

## Methods

### Study Design

This systematic review was conducted in accordance with the PRISMA (Preferred Reporting Items for Systematic Reviews and Meta-Analyses) 2020 guidelines (Checklist 1) [43]. For the development of the eligibility criteria, the SPIDER (sample, phenomenon of interest, design, evaluation, research type) framework [44] was applied to ensure a structured and targeted selection of studies.

### Eligibility Criteria

The eligibility criteria and methodological steps were defined a priori; however, no protocol was registered for this review. The inclusion and exclusion criteria were defined in alignment with the SPIDER components (Table 1), focusing for instance on studies involving nursing personnel in LTC (sample); their acceptance of digital technologies (phenomenon of interest); and empirical research designs with quantitative, qualitative, and mixed methods approaches (design and research type). This focus reflects the review’s aim to identify scientific evidence on how acceptance shapes digital adoption among LTC nursing staff. The studies had to be peer-reviewed and published in English or German. Exclusion criteria comprised studies conducted only in hospital, outpatient, or home care environments, as well as research focusing on other professional groups without separately analyzing the nursing staff perspective. Although qualitative and mixed methods studies were eligible according to the SPIDER framework, no such studies met all inclusion criteria (ie, focus on nursing staff in geriatric LTC and explicit assessment of technology acceptance). As a result, all included studies employed cross-sectional quantitative designs. This limitation is discussed in the *Results* and *Discussion* sections, but the inclusion parameters were retained to ensure methodological consistency and comparability across studies.

**Table 1.** Inclusion and exclusion criteria—SPIDER (sample, phenomenon of interest, design, evaluation, research type) components.

SPIDER components	Inclusion criteria	Exclusion criteria
S=Sample	Nursing staff employed in long-term care facilities (nursing homes, elderly care)	Studies focusing in general on non-nursing staff (eg, administrators, managers) Studies involving participants who are not working in long-term care facilities Studies with samples not clearly defined as nursing staff in geriatric long-term care
PI =Phenomenon of interest	Acceptance, adoption, barriers, experiences related to digital innovations in care settings, including technologies like electronic health records, telehealth services, assistive robotics, digital documentation, sensory, ICT^a^, IoT^b^, AI^c^-driven decision support systems	Studies focusing only on nondigitalized operations in long-term care Studies exclusively addressing competencies and education without looking at technology acceptance Studies not involving digital technologies
D=Design	Intervention studies, observational or cross-sectional surveys, studies employing qualitative, mixed methods designs	Nonresearch
E=Evaluation	Outcomes related to staff attitudes, perceptions, barriers, willingness to use, fears, and facilitators to adoption, satisfaction, perceived usefulness of digital technologies in long-term care	Studies not reporting on outcomes related to staff digital technology acceptance Studies focusing solely on managerial or administrative evaluations without staff input. Studies focusing only on nursing staff from hospitals or private home care settings
R=Research type	Qualitative, quantitative, or mixed methods research focusing on the care employees regarding digital innovation adoption Peer-reviewed journal articles published between January 1, 2010, and December 31, 2024 in English or German	Conference papers, reviews, editorials, letters to the editor, and studies not published in peer-reviewed journals Publications not in English or German Studies published outside the specified date range before January 1, 2010 (except for Yu et al [39])

a ICT: information and communication technology.

b IoT: internet of things.

c AI: artificial intelligence.

### Search Strategy

The search strategy employed an inclusive keyword combination, which was discussed and refined beforehand. Boolean operators were used to capture the intersection of acceptance, digitalization, technology, nursing, and geriatric LTC. The primary search string used was as follows: (“acceptance” AND (“digital technology” OR “digital” OR “technological” OR “artificial” OR “robotic” OR “digitalization” OR “artificial intelligence” OR “IoT” OR “robot” OR “virtual reality” OR “socially assistive robots” OR “digital tools” OR “telehealth” OR “Internet of Things” OR “EHR”)) AND (“nursing homes” OR “elderly” OR “geriatric” OR “inpatient home” OR “care facility” OR “nursing facilities” OR “nursing home” OR “aged care” OR “care home” OR “long-term care” OR “senior living center” OR “LTC”). Exact search strings for each database are documented in Multimedia Appendix 1.

The literature search was conducted across PubMed, Web of Science, ProQuest, and the ACM Digital Library. These databases were selected to ensure broad interdisciplinary coverage of nursing, health care, and technology-related research. Gray literature was not searched systematically. However, 1 relevant report identified through manual search [45] was used to provide contextual information for the discussion and was not part of the primary evidence base.

Although specialized databases, such as CINAHL, were not included due to missing license at University of Applied Sciences Neu-Ulm, the chosen databases offer considerable overlap. This limitation and the potential risk of missed studies are acknowledged in the *Discussion* section. To enhance comprehensiveness, the database search was supplemented by citation tracking and manual searches. Searches were limited to the period from January 1, 2010, to December 31, 2024. As noted previously, 1 of the included studies [39] falls outside the formal inclusion window set; however, it was retained based on discussions among all internal reviewers involved and due to the fact that this study represents the first known empirical study with the focus on the acceptance of digital technology among nursing staff in LTC settings. The identification process is illustrated in the PRISMA 2020 flow diagram, in the *Results* section.

The systematic search was conducted on April 14, 2025, following an initial exploratory search for an overview of the existing literature on October 25, 2024 (Multimedia Appendix 2). The primary researcher (JI) led the systematic review process, including database search, screening, and data extraction. The second reviewer (RH) independently screened the publications and also evaluated them for eligibility. Any discrepancies or critical assessments concerning study relevance, methodological quality, or thematic clarity were discussed in regular virtual meetings with senior reviewers (WS) and (DH). To manage the studies, the open-source software Zotero, version 7.0.11 (64-bit) was used as the reference software.

### Study Selection

Study selection was conducted in 2 phases. The first phase was the selection via title and reading the abstract. In the second phase, the full texts of potentially eligible studies were reviewed in detail. Studies that met the inclusion criteria and passed quality checks were included in the synthesis. Excluded studies and reasons for exclusion are presented in Multimedia Appendix 3.

Due to the limited number of eligible studies, a formal sensitivity analysis was not possible. However, the impact of study quality on synthesis outcomes was qualitatively assessed during reviewer discussions.

Data items from included studies were extracted with the following variables using an Excel form:

- Study identification: authors, title, year of publication, country
- Study design: methodological approach
- Participants: number, role (nursing staff, management)
- Aim of study: nature of the digital technology studied
- Key findings: outcome measures, determinants, and facilitators affecting acceptance

### Quality Assessment

Methodological quality was assessed using the Critical Appraisal Skills Programme (CASP) [46], applying item-level judgments (“Yes,” “Can’t tell,” “No”). The overall confidence rating was categorized as “low,” “moderate,” or “high,” with no studies falling into the “high” category. Each study was independently assessed by 2 reviewers across all checklist domains, including study aims, design, recruitment strategy, data collection, analysis, and potential bias. Discrepancies between the reviewers were resolved through consensus. To further strengthen methodological rigor and confirm the reliability of the CASP-based evaluations, the AXIS (Appraisal tool for Cross-Sectional Studies) [47] checklist for cross-sectional studies was additionally applied as a supplementary framework (Multimedia Appendix 4).

### Synthesis Approach

With regard to the synthesis approach, due to the heterogeneity of the included studies (technologies, outcome measures, countries), a narrative synthesis approach was applied keeping in mind the principles of thematic content analysis [48]. Data were coded inductively to identify recurring themes related to determinants and facilitators of digital technology acceptance. These themes were subsequently compared and mapped to ensure conceptual coherence across studies [49]. As this review analyzed previously published studies, no ethical approval was required.

## Results

### Study Selection

**Figure 1.**
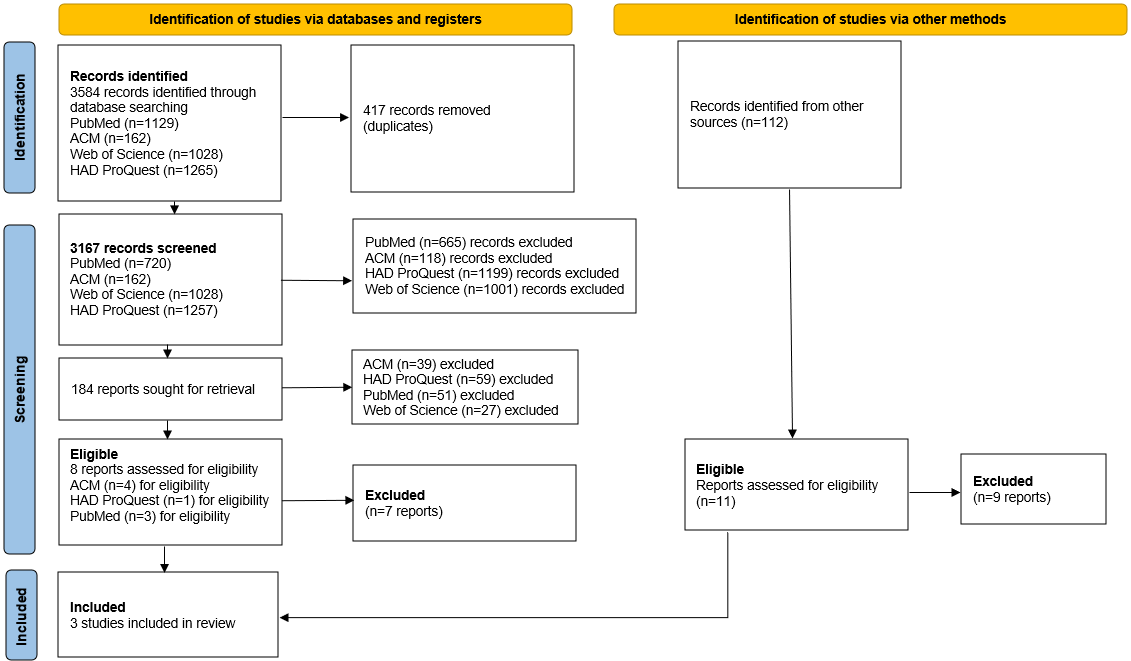
PRISMA (Preferred Reporting Items for Systematic Reviews and Meta-Analyses) 2020 flow diagram—identification of studies.

The outcome of the literature search initially yielded 3584 records from the databases and an additional 112 studies from citation tracking and manual searching as demonstrated in Figure 1.

After the removal of duplicates, the screening of the studies, and the application of eligibility criteria, 3 studies were included in the final analysis [12,26,39]. The included studies reported quantitative findings using the following measures:

- Likert-scale derived scores: these were used in all studies to assess acceptance variables (eg, attitudes, fears, perceived usefulness).
- Regression coefficients: these were reported in Barisch-Fritz et al [12] and Yu et al [39] to identify predictors of acceptance (eg, age, gender, professional group).
- Descriptive statistics: frequencies, means, and standard deviations were commonly used to present the results.

For this study, qualitative studies were eligible; however, none were identified for the final selection. Although limited in number, these studies offer initial insights into key acceptance factors and provide a basis for further investigation. These studies were conducted in Germany, the Netherlands, and Australia. Together, they involved 1019 participants, most of whom were direct care workers in nursing homes or LTC facilities. Across all studies, 867 were nursing staff, 99 were nursing home managers, and 53 were other staff members in LTC facilities (eg, clerks). The technologies under investigation ranged from electronic documentation systems to assistive robotic devices and digital communication platforms.

### Risk of Bias

The risk of bias was assessed using the CASP checklist for cross-sectional studies (Table 2). Individual checklist items were evaluated qualitatively to appraise methodological rigor, and each cell in Table 2 represents the reviewer’s consensus.

**Table 2.** Critical Appraisal Skills Programme (CASP) evaluation.

CASP	Barisch-Fritz et al (2023) [12]	de Veer et al (2011) [26]	Yu et al (2009) [39]
1. Did the study address a clearly focused issue?	Yes	Yes	Yes
2. Did the authors use an appropriate method to answer their question?	Yes	Yes	Yes
3. Were the subjects recruited in an acceptable way?	Yes	Yes	Cannot tell
4. Were the measures accurately measured to reduce bias?	Cannot tell	No	Yes
5. Were the data collected in a way that addressed the research issue?	Yes	Yes	Yes
6. Did the study have enough participants to minimize the play of chance?	Cannot tell	Yes	Cannot tell
7. How are the results presented and what is the main result?	Yes	Yes	Yes
8. Was the data analysis sufficiently rigorous?	Yes	Yes	Yes
9. Is there a clear statement of findings?	Yes	Yes	Yes
10. Can the results be applied to the local population?	Cannot tell	Yes	Cannot tell
11. Is the research valuable?	Cannot tell	Yes	Yes

While the CASP checklist provided a structured approach to appraising methodological quality, we also considered the AXIS critical appraisal tool, and it confirmed the initial CASP-based judgments.

Two studies [12,39] were rated as having moderate risk of bias. The study by Barisch-Fritz et al [12] addressed a clearly focused issue using validated instruments. Although the sample was good, it was not randomly selected, introducing potential self-selection bias. The study by Yu et al [39] also had a moderate risk of bias. It demonstrated strong internal validity through the use of validated TAM2-based instruments and a clearly defined research aim. However, some limitations remain; for instance, the convenience sampling reduced the strength of the recruitment process. Also, the relatively small sample size limits generalizability. These facts increase the potential for selection and sampling bias. The study by de Veer et al [26] demonstrated low risk of bias, supported by transparent reporting and robust measurement design. Nevertheless, the study lacked detailed information on how bias was addressed through measurement design.

### Study Characteristics

The key findings and characteristics of the included studies are summarized in Table 3. Across the 3 studies, several patterns emerged regarding the implementation and acceptance of technology in nursing and LTC settings. In the study by de Veer et al [26], approximately half of the nursing staff had encountered new technologies within the past 3 years and generally perceived these introductions positively. However, actual use was hindered by technology-related factors, such as ease of use, patient relevance, and potential risks. Respondents emphasized the need for structured innovation strategies and organizational support. Similarly, Yu et al [39] in Australia confirmed the validity of a modified TAM2 model for LTC facilities, identifying perceived usefulness, ease of use, professional image, and computer skills as primary determinants of the intention to adopt health IT applications. The German nationwide survey by Barisch-Fritz et al [12] extended these findings, showing that acceptance and technology affinity depend on education, professional role, and sociodemographic characteristics. Lower acceptance was observed among older employees.

**Table 3.** Included studies: key findings.

Authors	Title	Year of publication	Research method	Country	Aim of study	Which technology?	Participants included	Key findings
de Veer et al [26]	Successful implementation of new technologies in nursing care: a questionnaire survey of nurse-users	2011	Questionnaire survey	The Netherlands	To gain a better understanding of determinants influencing the success of the introduction of new technologies as perceived by nursing staff	New technologies introduced in the past three years Electronic information systems Distant care technology Medical devices	685 nursing staff	Half of the respondents were confronted with the introduction of new technology in the past 3 years Half of them rated the introduction of the technology as positive Factors impeding actual use were related to the technology itself: ie, malfunctioning, ease of use, relevance for patients, risk to patients Nursing staff stressed the importance of an adequate innovation strategy
Yu et al [39]	Health IT acceptance factors in LTC^a^ facilities: a cross-sectional survey	2009	Self-administered questionnaire	Australia	To examine the factors determining the acceptance of health IT applications by caregivers in LTC facilities	Health IT applications (software, documentation)	134 questionnaires Nurses (n=105) LTC clerks (n=11) Nursing managers (n=18)	Approved the validity of a modified TAM2^b^ in LTC facilities Factors influencing caregivers’ intention to use IT technology were perceived usefulness, perceived ease of use, image, and computer skills
Barisch-Fritz et al [12]	Are nursing home employees ready for the technical evolution? German-wide survey on the status quo of affinity for technology and technology interaction	2023	Online survey	Germany	Examine affinity for technology and technology interaction and related sociodemographic confounders, as well as detect possible requirements and boundary conditions relevant for the development and implementation of assistive technologies among nursing home employees	Technology, assistive technologies (eg, networked systems, assistive humanoid or social robots, mobile applications)	200 nursing home employees Nursing and therapy operation (n=77) Nursing home manager (n=81) Others in LTC (n=42)	Positive consequences depended on education and professional group and the affinity for technology varied across age and gender Lower acceptance with increasing age Lower acceptance for females Lower acceptance among nursing home managers

a LTC: long-term care.

b TAM2: technology acceptance model.

Despite differences in geographic context and methodological design, the studies share some overlapping findings regarding common factors that influence the acceptance. Perceived usefulness and perceived ease of use [26,39] consistently emerged as important determinants of acceptance. In addition, digital competence, defined as the ability to interact confidently with digital tools, was positively associated with willingness to use technology, particularly among younger staff members [12]. Organizational support, including leadership endorsement, training opportunities, and the involvement of staff in decision-making processes, also acted as a strong facilitator [26].

These cross-cutting themes are summarized in Table 4, which illustrates the main factors affecting the acceptance across studies.

**Table 4.** Factors affecting acceptance.

Authors	Title	Year of publication	Strengths	Weaknesses	Practical relevance	Factors affecting acceptance
de Veer et al [26]	Successful implementation of new technologies in nursing care: a questionnaire survey of nurse-users	2011	Strategic depth, very practical, multisectoral representativeness	Little quantitative analysis Mainly qualitative; not 100% LTC-specific^a^	Very high: helpful for implementation planning, LTC sector, and hospital	Involvement of nursing staff during development and implementation affects acceptance. Organizational support, such as leadership endorsement, communication, and available training does increase adoption. Perceived relevance of the technology for patient care enhances likelihood of use.
Yu et al [39]	Health IT acceptance factors in LTC facilities: a cross-sectional survey	2009	Theoretically grounded, structural modeling, clear implications	Limited representativeness, convenience sample, preimplementation data	Moderate to high: theoretical insights; limited practical transferability relevant for IT strategies in the LTC context	Perceived usefulness is the strongest predictor of care staff intention to use digital technologies. Digital competence correlates positively with willingness to use technology, particularly among younger staff. Negative perceptions through IT use (image factor) reduce acceptance. Ease of use significantly influences both perceived usefulness and intention to adopt technology. Perceived relevance of the technology for patient care enhances likelihood of use.
Barisch-Fritz et al [12]	Are nursing home employees ready for the technical evolution? German-wide survey on the status quo of affinity for technology and technology interaction	2023	Good sample, valid measurement instruments, differentiated results	Confounder control Nonrandom sampling, response bias likely	High: directly applicable to nursing homes	Digital competence correlates positively with willingness to use technology, particularly among younger staff. Technology affinity varies strongly across age, gender, and professional role. Organizational support, such as leadership endorsement, communication, and available training does increase adoption. Ethical concerns can limit technology acceptance.

a LTC: long-term care.

All 3 studies contributed important evidence regarding factors influencing acceptance, organizational support, training availability, perceived usefulness, and digital competence. To account for heterogeneity across technologies and study designs, the extracted data were grouped thematically into 3 analytical levels: individual, organizational, and technological (Table 5).

This comparative thematic structure enabled a coherent synthesis across diverse contexts. Perceived usefulness, digital competence, organizational readiness, and usability emerged consistently across studies, supporting central constructs of the TAM.

**Table 5.** Thematic synthesis of factors affecting acceptance.

Level	Technology type	Emerging themes	Example evidence	Studies contributing
Individual	Electronic information and documentation systems; telecare software	Digital literacy, perceived usefulness, professional image, computer self-efficacy	Staff with higher digital competence and positive attitudes toward electronic documentation and telecare reported higher acceptance. Perceived usefulness and ease of use predicted intention to adopt these systems.	Barisch-Fritz et al (2023) [12] ; Yu et al (2009) [39]
Organizational	EHR^a^ systems; digital readiness tools	Training, managerial support, workload, innovation climate	Organizational readiness, management involvement, and access to training facilitated technology use, while workload and lack of structured implementation strategies reduced uptake.	de Veer et al (2011) [26]; Barisch-Fritz et al (2023) [12]
Technological	Assistive technologies; robots; health IT software	Usability, reliability, system relevance, perceived ethical and professional implications	Usability and reliability were decisive for acceptance across all technologies, whereas assistive and robotic technologies introduced concerns regarding trust, ethics, and role identity.	de Veer et al (2011) [26]; Yu et al (2009) [39]; Barisch-Fritz et al (2023) [12]

a EHR: electronic health record.

## Discussion

### Interpretation of Findings

The synthesis of the 3 studies revealed that the acceptance of digital technologies in geriatric LTC depended on a combination of individual and organizational factors. Consistent with TAM and its extensions, usefulness and ease of use were the most robust predictors across the studies.

Beyond individual and organizational determinants, contextual factors, such as organizational culture, leadership style, and national policy frameworks, also influence digital readiness in LTC. Environments with a long-standing emphasis on innovation and participatory care culture may facilitate staff involvement in digital implementation, whereas strict data-protection orientation and reliance on paper-based processes may hinder the change. National eHealth infrastructures, such as Germany’s Telematics Infrastructure and reimbursement policies, can affect incentives for adoption. Recognizing these dimensions is essential, as technological acceptance should not be understood in isolation from broader policy and organizational environments [50].

Previous reviews have also highlighted the importance of user attitudes and digital competencies for successful implementation [51-53]. Staff who feel confident in their ability to use digital tools are more willing to adopt them. This is particularly relevant given the generational differences observed in digital affinity. Younger staff members tend to have higher levels of acceptance, while older staff may require more training and support.

Organizational conditions further contribute to acceptance. Early staff involvement in the selection, testing, and implementation of new technologies, combined with training and transparent communication, fosters adoption.

A valuable complement to the peer-reviewed evidence is the BGW report “Pflege 4.0” [45], which constitutes gray literature but offers important contextual insights. Drawing on a mixed methods dataset of 576 professional caregivers in Germany—140 of whom were from geriatric LTC facilities—the report explored both actual technology use and perceived barriers to adoption. Using various 5-point Likert scales (ranging from “does not apply” to “fully applies”; from “not familiar at all” to “very familiar”), the survey identified key concerns, such as fear of job loss, data protection concerns, lack of technical skills, and low participation in implementation processes. While the professional composition of respondents was not fully specified, the findings add practical relevance by highlighting workplace-level perceptions that mirror those reported in the peer-reviewed studies.

### Limitations of Evidence

The limited number (n=3) of eligible studies and their predominantly cross-sectional nature restrict the ability to draw clear conclusions, even though they identify relevant influencing factors. Additionally, the studies differ in the types of technologies investigated, outcome measures used, representation of demographic groups, and regional contexts. This heterogeneity complicates direct comparisons, and it further limits the generalizability of the findings. For instance, Yu et al [39] conducted a preimplementation survey based on TAM2 in an Australian LTC context, focusing on intention to use the technology. On the other hand, de Veer et al [26] investigated actual technology implementation across multiple health care sectors in the Netherlands, including nursing homes, but not exclusively. Barisch-Fritz et al [12] explored technology affinity in German nursing homes, but their heterogeneous sample included managers and other staff in LTC facilities with a relatively small response rate. This fact raises concerns regarding representativeness. These limitations hinder generalizability.

Although comprehensive efforts were made to include all relevant research, the review was limited to publications in English or German, and no protocol was registered in advance. In addition, the CINAHL database was not searched due to a missing institutional license. As CINAHL is a relevant source, other studies may not have been captured.

### Implications

Given the limited number of studies and their methodological heterogeneity, the implications hereby should be interpreted with caution. Nevertheless, the evidence indicates that the successful implementation of digital technologies in geriatric LTC relies on strategies that are aligned to the needs, competencies, and experiences of nursing staff. Policies should prioritize ongoing digital training programs based on the different groups of users. Furthermore, implementation efforts should involve staff from the earliest planning stages, ensuring that their expertise informs both system design and rollout. Organizational support and transparent communication regarding the objectives, benefits, and limitations of new systems are essential to build trust and reduce uncertainty among nursing staff. Ethical concerns must be addressed proactively, particularly in relation to surveillance technologies and the preservation of interpersonal care dynamics. In terms of research, there is definitely a need for more robust, rigorous, and longitudinal studies to enhance external validity and provide a more comprehensive understanding of technology acceptance among nursing staff in geriatric LTC.

### Conclusion

This systematic review demonstrates that the acceptance of digital technologies by nursing staff in geriatric LTC settings is shaped by a constellation of individual and organizational factors. Three key determinants emerged consistently across all studies.

First, digital competence significantly influences willingness to adopt new technologies. Nursing staff with higher digital affinity, especially younger staff members, show greater readiness to engage with digital tools in the workplace. This highlights the need for training programs that target all age and experience groups.

Second, perceived relevance of technologies to daily care practice affects acceptance. Nursing staff are more likely to accept innovations that support main aspects of nursing home care, such as documentation efficiency, communication, or safety.

Third, organizational support, including communication, managerial encouragement, access to training, and staff participation in the implementation processes of digital technologies, plays a crucial role.

In light of the structural and demographic relevance of geriatric LTC, future research should be directed toward building a strong evidence base on technology acceptance. This review offers several testable hypotheses derived from the synthesized evidence. Future studies should empirically examine how early involvement of nursing staff in the development and implementation of digital technologies affects subsequent acceptance and sustained use. It can be hypothesized that organizational support mechanisms, including leadership endorsement, effective communication, and targeted digital training, strengthen the relationship between perceived usefulness and intention to use. Likewise, digital competence may mediate the relationship between training and technology adoption, while factors such as technology affinity, age, and professional role may moderate these effects. Furthermore, perceived relevance for patient care likely increases acceptance by reinforcing the perceived usefulness of digital tools, whereas ethical concerns or a negative professional image of IT use may inhibit adoption. Testing these mechanisms through longitudinal, intervention-based, or multilevel study designs could provide stronger causal evidence for the transformation strategies in geriatric LTC.

## Supplementary material

10.2196/82223 Multimedia Appendix 1 Search strings for the databases searched.

10.2196/82223 Multimedia Appendix 2 Overview search in PubMed conducted on October 25, 2025, to explore the topic prior to the systematic search.

10.2196/82223 Multimedia Appendix 3 Excluded publications after eligibility assessment according to the exclusion criteria.

10.2196/82223 Multimedia Appendix 4 AXIS (Appraisal tool for Cross-Sectional Studies) quality assessment of included studies.

10.2196/82223 Checklist 1 PRISMA 2020 checklist.

## Abbreviations

AXIS: Appraisal tool for Cross-Sectional Studies
CASP: Critical Appraisal Skills Programme
LTC: long-term care
PRISMA: Preferred Reporting Items for Systematic Reviews and Meta-Analyses
SPIDER: sample, phenomenon of interest, design, evaluation, research type
TAM: technology acceptance model
