# Supplementary figures and images for: Acceptance of Digital Technology Among Nursing Staff in Geriatric Long-Term Care: Systematic Review
Source: JMIR Nurs. 2026 Jan 15;9:e82223. doi: 10.2196/82223 (PMC12807401; doi:10.2196/82223)

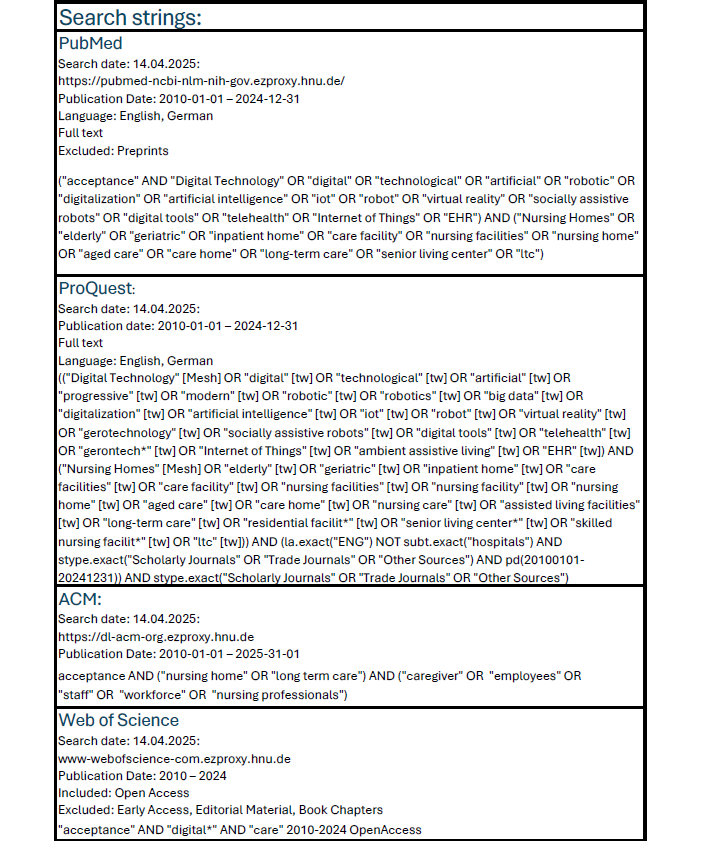

Supplement: Multimedia Appendix 1 [file nursing-v9-e82223-s001.png]

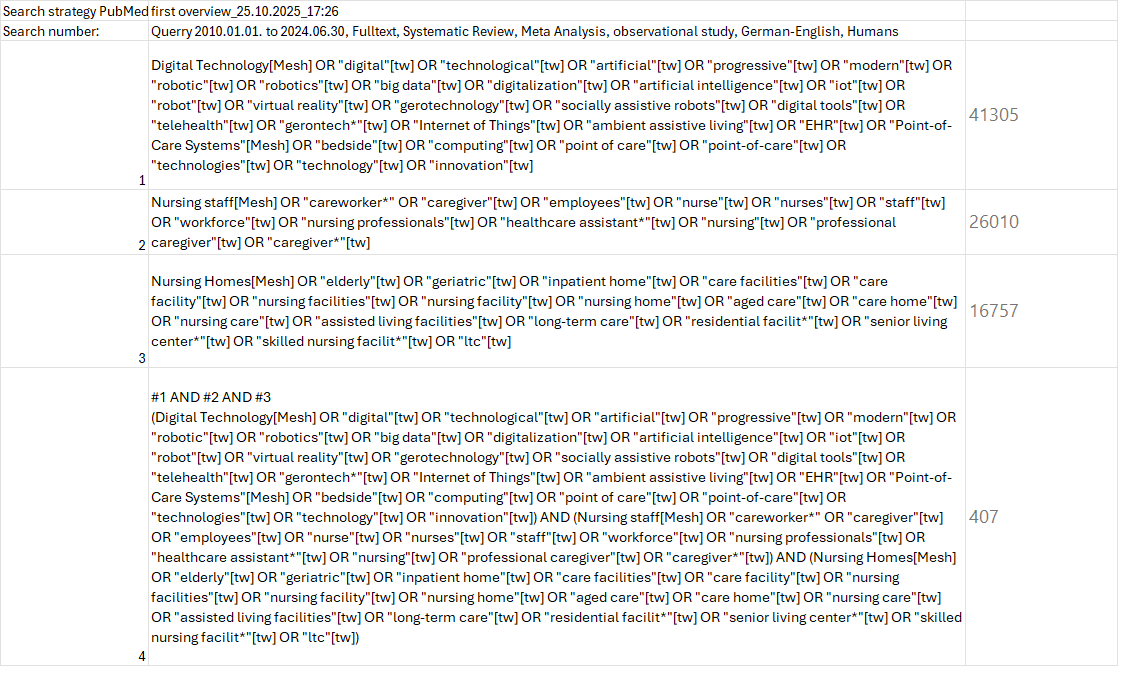

Supplement: Multimedia Appendix 2 [file nursing-v9-e82223-s002.png]
